# Complete Genome Sequence of Salmonella enterica Serovar Enteritidis Siphophage Seafire

**DOI:** 10.1128/MRA.01167-19

**Published:** 2019-10-24

**Authors:** Sarah Hartman, Chi Zeng, Chandler O’Leary, Heather Newkirk, Rohit Kongari, Jason Gill, Mei Liu

**Affiliations:** a Center for Phage Technology, Texas A&M University, College Station, Texas, USA; b School of Biology and Pharmaceutical Engineering, Wuhan Polytechnic University, Wuhan, China; DOE Joint Genome Institute

## Abstract

Bacteriophages infecting Salmonella enterica subsp. *enterica* serovar Enteritidis may be used as biocontrol agents in food products or animals for preventing foodborne diseases caused by this pathogen. The complete genome sequence of phage Seafire, a T5-like siphophage infecting *S.* Enteritidis, is described in this report.

## ANNOUNCEMENT

The Gram-negative bacterium Salmonella enterica subsp. *enterica* serovar Enteritidis is a major cause of gastroenteritis in humans, resulting from the consumption of contaminated eggs or undercooked poultry meat (1). Salmonella infection is characterized by its ability to invade and colonize host epithelial cells (2). The rise of multidrug resistance among *Salmonella* strains makes phage therapy an attractive control method for this bacterium (3, 4). Here, we present the complete genome sequence of Seafire, a bacteriophage capable of infecting *S.* Enteritidis.

Siphophage Seafire was isolated from a wastewater treatment plant in College Station, TX, in 2015, using a poultry isolate of *S.* Enteritidis as the host. Host bacteria were cultured on tryptic soy broth or agar (Difco) at 37°C with aeration. Phages were cultured and propagated by the soft-agar overlay method (5). The phage was identified as a siphophage using negative-stain transmission electron microscopy performed at the Texas A&M University Microscopy and Imaging Center as described previously (6). Phage genomic DNA was prepared using a modified Promega Wizard DNA cleanup kit protocol (6). Pooled indexed DNA libraries were prepared using the Illumina TruSeq Nano LT kit, and the sequence was obtained with the Illumina MiSeq platform using the MiSeq v2 500-cycle reagent kit following the manufacturer’s instructions, producing 605,092 paired-end reads for the index containing the phage Seafire genome. FastQC v0.11.5 (https://www.bioinformatics.babraham.ac.uk/projects/fastqc/) was used for quality control of reads. The reads were trimmed with FastX Toolkit v0.0.14 (http://hannonlab.cshl.edu/fastx_toolkit/download.html) before being assembled using SPAdes v3.5.0 (7). Contig completion was confirmed by PCR using primers (5′-ACATGATGGACAGCGTGGT-3′ and 5′-GGCACTTTCTCATCAACAACAA-3′) oriented toward the ends of the assembled contig and by Sanger sequencing of the resulting product, with the contig sequence manually corrected to match the resulting Sanger sequencing read. GLIMMER v3.0 (8) and MetaGeneAnnotator v1.0 (9) were used with manual verification to predict protein coding genes, and tRNA genes were predicted with ARAGORN v2.36 (10). Rho-independent termination sites were identified via TransTermHP (http://transterm.cbcb.umd.edu/). Sequence similarity searches were done by BLASTp v2.2.28 (11) with a maximum expectation cutoff of 0.001 against the NCBI non-redundant (nr), UniProt Swiss-Prot (12), and TrEMBL databases. InterProScan v5.15-54.0 (13), LipoP (14), and TMHMM v2.0 (15) were used to predict protein function. HHpred with ummiclust30_2018_08 for multiple sequence alignment (MSA) generation and PDB_mmCIF70 for modeling in the HHsuite v3.0 release were also used for functional prediction (16). All analyses were conducted using default settings via the CPT Galaxy (17) and WebApollo (18) interfaces (https://cpt.tamu.edu/galaxy-pub).

Phage Seafire was assembled at 27.8-fold coverage to a single contig of 111,851 bp containing only one copy of a 9,592-bp direct terminal repeat determined by PhageTerm (19). Seafire exhibits a GC content of 40.0%. Seafire shares 87% and 61% overall sequence identity with *Salmonella* phages Stitch (GenBank accession no. KM236244) (20) and T5 (GenBank accession no. AY543070), respectively, as determined by BLASTn. T5-like gene clusters (21) were identified in Seafire. These include the pre-early region proteins (A1, A2, and deoxynucleoside-5′-monophosphatase) involved in the first step of transfer of injected DNA (22); the early region proteins involved in DNA replication, repair, and metabolism; and the late region proteins involved in host cell lysis (holin, endolysin, and a spanin pair) and phage assembly.

### Data availability.

The genome sequence of phage Seafire was submitted to GenBank under accession no. MK050846. The associated BioProject, SRA, and BioSample accession numbers are PRJNA222858, SRR8771449, and SAMN11233789, respectively.
